# Prostate Cancer Risk Calculator Apps in a Taiwanese Population Cohort: Validation Study

**DOI:** 10.2196/16322

**Published:** 2020-12-18

**Authors:** I-Hsuan Alan Chen, Chi-Hsiang Chu, Jen-Tai Lin, Jeng-Yu Tsai, Chia-Cheng Yu, Ashwin Narasimha Sridhar, Prasanna Sooriakumaran, Rui C V Loureiro, Manish Chand

**Affiliations:** 1 Division of Urology Department of Surgery Kaohsiung Veterans General Hospital Kaohsiung Taiwan; 2 School of Medicine National Yang-Ming University Taipei Taiwan; 3 Division of Urology, Department of Surgery Tri-Service General Hospital National Defense Medical Center Taipei Taiwan; 4 Division of Surgery and Interventional Sciences University College London London United Kingdom; 5 Department of Statistics National Cheng Kung University Tainan Taiwan; 6 Department of Uro-Oncology University College London Hospital London United Kingdom; 7 Nuffield Department of Surgical Sciences University of Oxford Oxford United Kingdom; 8 Aspire Centre for Rehabilitation Engineering and Assistive Technology University College London and the Royal National Orthopaedic Hospital Stanmore United Kingdom; 9 Department of Colorectal Surgery University College London Hospital London United Kingdom

**Keywords:** diagnosis, mHealth, mobile apps, prostate cancer, prostate-specific antigen, risk calculator

## Abstract

**Background:**

Mobile health apps have emerged as useful tools for patients and clinicians alike, sharing health information or assisting in clinical decision-making. Prostate cancer (PCa) risk calculator mobile apps have been introduced to assess risks of PCa and high-grade PCa (Gleason score ≥7). The Rotterdam Prostate Cancer Risk Calculator and Coral–Prostate Cancer Nomogram Calculator apps were developed from the 2 most-studied PCa risk calculators, the European Randomized Study of Screening for Prostate Cancer (ERSPC) and the North American Prostate Cancer Prevention Trial (PCPT) risk calculators, respectively. A systematic review has indicated that the Rotterdam and Coral apps perform best during the prebiopsy stage. However, the epidemiology of PCa varies among different populations, and therefore, the applicability of these apps in a Taiwanese population needs to be evaluated. This study is the first to validate the PCa risk calculator apps with both biopsy and prostatectomy cohorts in Taiwan.

**Objective:**

The study’s objective is to validate the PCa risk calculator apps using a Taiwanese cohort of patients. Additionally, we aim to utilize postprostatectomy pathology outcomes to assess the accuracy of both apps with regard to high-grade PCa.

**Methods:**

All male patients who had undergone transrectal ultrasound prostate biopsies in a single Taiwanese tertiary medical center from 2012 to 2018 were identified retrospectively. The probabilities of PCa and high-grade PCa were calculated utilizing the Rotterdam and Coral apps, and compared with biopsy and prostatectomy results. Calibration was graphically evaluated with the Hosmer-Lemeshow goodness-of-fit test. Discrimination was analyzed utilizing the area under the receiver operating characteristic curve (AUC). Decision curve analysis was performed for clinical utility.

**Results:**

Of 1134 patients, 246 (21.7%) were diagnosed with PCa; of these 246 patients, 155 (63%) had high-grade PCa, according to the biopsy results. After confirmation with prostatectomy pathological outcomes, 47.2% (25/53) of patients were upgraded to high-grade PCa, and 1.2% (1/84) of patients were downgraded to low-grade PCa. Only the Rotterdam app demonstrated good calibration for detecting high-grade PCa in the biopsy cohort. The discriminative ability for both PCa (AUC: 0.779 vs 0.687; DeLong’s method: *P*<.001) and high-grade PCa (AUC: 0.862 vs 0.758; *P*<.001) was significantly better for the Rotterdam app. In the prostatectomy cohort, there was no significant difference between both apps (AUC: 0.857 vs 0.777; *P*=.128).

**Conclusions:**

The Rotterdam and Coral apps can be applied to the Taiwanese cohort with accuracy. The Rotterdam app outperformed the Coral app in the prediction of PCa and high-grade PCa. Despite the small size of the prostatectomy cohort, both apps, to some extent, demonstrated the predictive capacity for true high-grade PCa, confirmed by the whole prostate specimen. Following our external validation, the Rotterdam app might be a good alternative to help detect PCa and high-grade PCa for Taiwanese men.

## Introduction

The use of health-related apps is increasing within health care systems. Prostate cancer (PCa) risk calculator mobile apps have been introduced to assess risks of PCa and high-grade PCa (Gleason score ≥7). The Rotterdam Prostate Cancer Risk Calculator and Coral–Prostate Cancer Nomogram Calculator apps were developed from the 2 most-studied PCa risk calculators, the European Randomized Study of Screening for Prostate Cancer (ERSPC) [1] and the North American Prostate Cancer Prevention Trial (PCPT) [2] risk calculators, respectively. Adam et al [3] performed a critical appraisal of 7 PCa risk calculator apps, indicating that the Rotterdam and Coral apps performed best during the prebiopsy stage. According to the currently available evidence, both apps have only been externally validated by a 2-center European study. They have demonstrated better predictive accuracy than prostate-specific antigen (PSA) and digital rectal examination (DRE) [4].

In Taiwan, around 40% of new PCa cases are diagnosed as locally advanced or metastatic diseases, which is less favorable than the stage distribution of Western countries [5]. This has not changed remarkably over the last two decades, albeit the incidence of PCa has been increasing in Taiwan since 1979. Metastatic PCa still made up almost 30% of newly-diagnosed cases from 2004 to 2012 compared to a proportion of 32.7% from 1977 to 1997 [6]. For early detection of PCa, risk calculator apps may help assess the risk of PCa or high-grade PCa in the Taiwanese population. Moreover, with the capacity to differentiate high-grade PCa, active surveillance might be supported by these apps during patient counseling.

The aim of this study is to evaluate the performance of PCa risk calculator apps in a Taiwanese population. We performed external validation using a Taiwanese cohort of patients who had undergone transrectal ultrasound (TRUS) prostate biopsy. Additionally, in previous validation studies for PCa risk calculators or apps, risk stratification was based on biopsy outcomes instead of postprostatectomy pathology results. Accordingly, we aimed to utilize postprostatectomy pathology outcomes to assess the accuracy of both apps with regard to high-grade PCa.

## Methods

### Inclusion Criteria

Internal review board approval (IRB No.: VGHKS19-CT3-13) was granted by a Taiwanese tertiary medical center, the Kaohsiung Veterans General Hospital. All male patients (N=1344) undergoing TRUS prostate biopsies with a 12-core systematic biopsy strategy from 2012 to 2018 were enrolled. The indication for prostate biopsy included an abnormal PSA level (>4 ng/mL) or an abnormal DRE. Each patient would receive DRE and TRUS before the biopsy was performed; prostate volume (PV) was calculated by the ellipsoid formula (length x width x height x π/6). Some patients (53/1344) had multiparametric magnetic resonance imaging (mpMRI) scans on a self-pay basis because the Taiwan National Health Insurance system has not approved the reimbursement of pelvic magnetic resonance imaging (MRI) before prostate biopsy. All prebiopsy mpMRI scans were reported by dedicated urologic radiologists, in agreement with the Prostate Imaging Reporting and Data System, version 2 (PI-RADS v2) [7]. The number of patients who received radical prostatectomy was 137. Consultant pathologists reviewed all biopsies and postprostatectomy specimens.

According to the Rotterdam app, the definition of clinically significant PCa is a tumor stage greater than T2b, or a Gleason biopsy score of ≥7, which is identical to high-risk PCa in the ERSPC risk calculator (ERSPC-RC) [8]. In comparison, the Coral app defined a Gleason biopsy score of ≥7 as high-grade PCa, which originated from the PCPT [2]. In order to use consistent terminology, a Gleason score of ≥7 was defined as high-grade PCa. PSA was designated as the latest total serum PSA level before prostate biopsy.

### Data Collection

All patient data were retrospectively collected via electronic medical records. The Rotterdam app accepts input data on age, DRE history and outcome, previous negative biopsy, PSA, PV, volume measure method (TRUS or DRE), TRUS evaluation (normal or abnormal), MRI history, and PI-RADS score. The Coral app requires data on ethnicity (African American, Caucasian, Hispanic, or Other), age, DRE, PSA, family history, and prior biopsy results. Following input data collection, the risks of PCa and high-grade PCa were calculated using the Rotterdam and Coral apps.

### Exclusion Criteria

**Figure 1:**
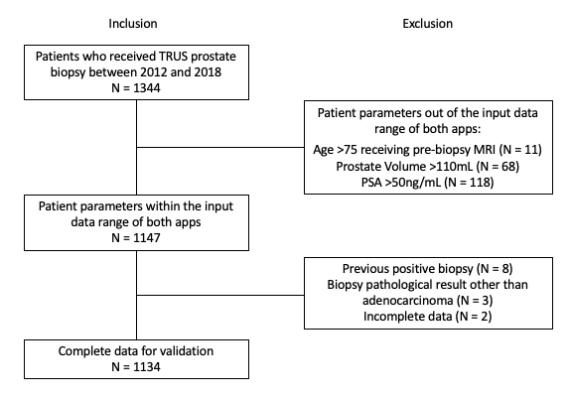
Flowchart of patient enrollment into the study. MRI: magnetic resonance imaging; PSA: prostate-specific antigen; TRUS: transrectal ultrasound.

Each app customizes its parameters to impose controls or constraints on accepted input values; that is, both the Rotterdam and Coral apps have an input data range for some parameters. For instance, the input PSA range is limited from 0.4-50 ng/mL within the Rotterdam app and 0.3-100 ng/mL within the Coral app. If the patients have prebiopsy MRI scans, there is an age limit between 50 and 75 years. On the contrary, without prebiopsy MRI, both the Rotterdam and Coral apps have no limitation on the input age data. According to the accepted input values from both apps, 197 patients were excluded, either because their PV was <10 or >110 mL (68/197), or their PSA level was <0.4 or >50 ng/mL (118/197), or they underwent prebiopsy MRI at the age of <50 or >75 years (11/197). In addition, 13 more patients with previous positive biopsies (8/13), pathological diagnosis different from adenocarcinoma (3/13), or incomplete data (2/13) were excluded. Details of the inclusion and exclusion process are illustrated in the flow chart in Figure 1.

### Statistical Analysis

Statistical analyses were performed utilizing SPSS (version 18; IBM Corp) and R software packages (R Core Team). The Kolmogorov-Smirnov test was used to examine the normality of the distribution of variables. Categorical variables were assessed with the chi-square test. Continuous variables were described as medians and interquartile ranges or means and standard deviations, and compared by the Mann-Whitney *U* test or the Student independent *t* test based upon their nonnormal or normal distributions, respectively. The applicability of each PCa risk calculator app in the Taiwanese population cohort was statistically analyzed on the basis of its discrimination, calibration, and clinical utility [9]. Calibration relates to the agreement between the observed and predicted proportion of events; calibration was evaluated graphically utilizing a calibration plot in which the observed probabilities were plotted against the predicted probabilities, enabling assessment of the extent of risk underestimation or overestimation [10]. The statistical significance of miscalibration was examined by the Hosmer-Lemeshow goodness-of-fit test [11].

Discrimination reflects the capacity of a prediction model to differentiate between those with and without an event (any-grade or high-grade PCa) and is quantified utilizing the area under the receiver operating characteristic (ROC) curve (AUC). The AUCs of the Rotterdam and Coral apps were compared using DeLong’s method [12]. As for clinical utility, decision curve analysis was performed to analyze whether both apps were beneficial for clinical decision-making or which app would lead to better decisions. We calculated the net benefit to quantify the clinical utility; different threshold probabilities mean different harm-to-benefit ratios. Net benefit was formulated as the number of true positives subtracted from the proportion of false positives weighted by the odds of the risk threshold probability, and the result was divided by the sample size. By measuring the proportions of *net* true positives in the models, we could assess whether any model performed better than others and the default strategies of biopsying all or no patients across the reasonable range of risk threshold probabilities [13].

## Results

### Patient Demographics

Of 1344 patients undergoing biopsies, 246 (21.7%) patients were diagnosed with PCa; of these 246 patients, 155 (63%) had high-grade PCa, according to the biopsy results. Compared to males with negative biopsies, patients with PCa were significantly older, had higher PSA levels, smaller PVs, more abnormal findings on TRUS and DRE, and higher PI-RADS scores demonstrated on mpMRI (Table 1). Both in the biopsy and prostatectomy cohorts, patients with high-grade PCa had significantly higher PSA, lower PV, and more abnormal findings on TRUS compared to those with low-grade PCa (Table 2). Among 246 diagnoses of PCa, 137 patients underwent radical prostatectomy; based on the postprostatectomy outcomes, 47.2% (25/53) of patients were upgraded to high-grade PCa and 1.2% (1/84) of patients were downgraded to low-grade PCa.

**Table 1:** Patient demographics (N=1344). Categorical variables were assessed with the chi-square test; continuous variables were compared by the Mann-Whitney U test or the Student independent t test based on their nonnormal or normal distribution, respectively.

Characteristics	All patients (n=1134)	Patients with no cancer (n=888, 78.3%)	Patients with cancer (n=246, 21.7%)	*P* value
Age in years, mean (SD); median (1^st^ quartile-3^rd^ quartile)	68.78 (8.23); 67 (61-73)	66.31 (8.17); 66 (61-72)	68.49 (8.25); 69 (63-74)	<.001
PSA^a^, mean (SD); median (1^st^ quartile-3^rd^ quartile)	10.27 (7.44); 8.08 (5.73-11.65)	9.20 (5.90); 7.66 (5.57-10.44)	14.12 (10.53); 10.16 (6.64-17.51)	<.001
PV^b^, mean (SD); median (1^st^ quartile-3^rd^ quartile)	52.72 (21.13); 48.35 (37.08-66.01)	55.35 (20.86); 51.52 (39.93-68.88)	43.25 (19.36), 37.82 (29.68-52.00)	<.001
Family history, n (%)	44 (3.9)	32 (3.6)	12 (4.9)	.360
Suspicious TRUS^c^, n (%)	253 (22.3)	151 (17.0)	102 (41.5)	<.001
Suspicious DRE^d^, n (%)	192 (16.9)	89 (10.0)	103 (41.9)	<.001
MRI^e^ (n=38) PI-RADS^f^ 4,5, n (%)	27 (71.1)	14 (58.3)	13 (92.9)	.030
Rotterdam PCa^g^(%), mean (SD); median (1^st^ quartile-3^rd^ quartile)	29.98 (23.98); 21 (13-39)	23.92 (17.69); 18 (12-31)	51.84 (30.20); 50 (24-84)	<.001
Rotterdam high-grade PCa (%), mean (SD); median (1^st^ quartile-3^rd^ quartile)	14.30 (21.47); 5 (2-14)	8.66 (12.64); 4 (2-9)	34.60 (31.98); 21 (6-65)	<.001
Coral PCa (%), mean (SD); median (1^st^ quartile-3^rd^ quartile)	34.37 (11.53); 32 (26-39)	32.42 (9.64); 31 (26-36)	41.42 (14.63); 38 (31.00-51.25)	<.001
Coral high-grade PCa (%), mean (SD); median (1^st^ quartile-3^rd^ quartile)	14.59 (10.38); 11 (8-18)	12.74 (8.05); 11 (7-16)	21.24 (14.38); 17 (10-30)	<.001

^a^PSA: prostate-specific antigen.

^b^PV: prostate volume.

^c^TRUS: transrectal ultrasound.

^d^DRE: digital rectal examination.

^e^MRI: magnetic resonance imaging.

^f^PI-RADS: Prostate Imaging Reporting and Data System.

^g^PCa: prostate cancer.

**Table 2:** Demographics of patients with prostate cancer (PCa; n=383). Categorical variables were assessed with the chi-square test; continuous variables were compared by the Mann-Whitney U test or the Student independent t test based on their nonnormal or normal distribution, respectively.

Characteristics	Biopsy cohort (n=246)			Prostatectomy cohort (n=137)		
	Low-grade PCa^a^ (n=91, 37%)	High-grade PCa (n=155, 63%)	*P* value	Low-grade PCa (n=29, 21%)	High-grade PCa (n=108, 79%)	*P* value
Age in years, mean (SD)	67.22 (8.00)	69.24 (8.33)	.064	63.07 (7.21)	65.79 (6.27)	.047
PSA^b^, mean (SD); median (1^st^ quartile-3^rd^ quartile)	9.94 (7.36); 7.9 (5.1-12.2)	16.57 (11.33); 13.0 (8.1-23.2)	<.001	6.68 (2.60); 7.1 (4.7-8.4)	14.33 (10.27); 11.1 (6.7-17.9)	<.001
PV^c^, mean (SD); median (1^st^ quartile-3^rd^ quartile)	47.56 (22.30); 42.4 (30.4-59.7)	40.71(16.97); 36.4 (28.9-48.9)	.039	51.53 (22.13); 46.6 (32.5-61.0)	39.80 (17.26); 34.2 (28.6-46.3)	.004
Family history, n (%)	4 (4.4)	8 (5.2)	>.99	1 (3.4)	7 (6.5)	>.99
Suspicious TRUS^d^, n (%)	18 (19.8)	84 (54.2)	<.001	1 (3.4)	41 (38.0)	<.001
Suspicious DRE^e^, n (%)	27 (29.7)	76 (49.0)	.003	7 (24.1)	45 (41.7)	.084
MRI^f^ (n=14) PI-RADS^g^ 4,5, n (%)	2 (100.0)	11 (91.7)	>.99	-	10 (90.9)	-
Rotterdam PCa (%), mean (SD); median (1^st^ quartile-3^rd^ quartile)	35.13 (26.39); 25.0 (15.0-54.0)	61.65 (27.96); 66.0 (36.0-88.0)	<.001	21.72 (14.70); 18.0 (12.5-25.0)	54.32 (27.91); 52.0 (28.3-84.0)	<.001
Rotterdam high-grade PCa (%), mean (SD); median (1^st^ quartile-3^rd^ quartile)	18.74 (25.72); 7.0 (3.0-21.0)	43.92 (31.69); 36.0 (15.0-75.0)	<.001	7.14 (8.50); 4.0 (3.0-7.0)	34.39 (28.93); 23.5 (10.0-60.3)	<.001
Coral PCa (%), mean (SD); median (1^st^ quartile-3^rd^ quartile)	35.11 (11.86); 31.0 (26.0-41.0)	45.13 (14.86); 42.0 (34.0-55.0)	<.001	28.34 (6.14); 27.0 (24.0-34.0)	39.43 (12.29); 37.0 (30.0-48.0)	<.001
Coral high-grade PCa (%), mean (SD); median (1^st^ quartile-3^rd^ quartile)	15.37 (10.71); 12.0 (8.0-19.0)	24.68 (15.16); 20.0 (13.0-34.0)	<.001	9.48 (4.99); 8.0 (6.0-13.5)	18.92 (11.55); 15.0 (10.0-25.0)	<.001

^a^PCa: prostate cancer.

^b^PSA: prostate-specific antigen.

^c^PV: prostate volume.

^d^TRUS: transrectal ultrasound.

^e^DRE: digital rectal examination.

^f^MRI: magnetic resonance imaging.

^g^PI-RADS: Prostate Imaging Reporting and Data System.

### Calibration

**Figure 2:**
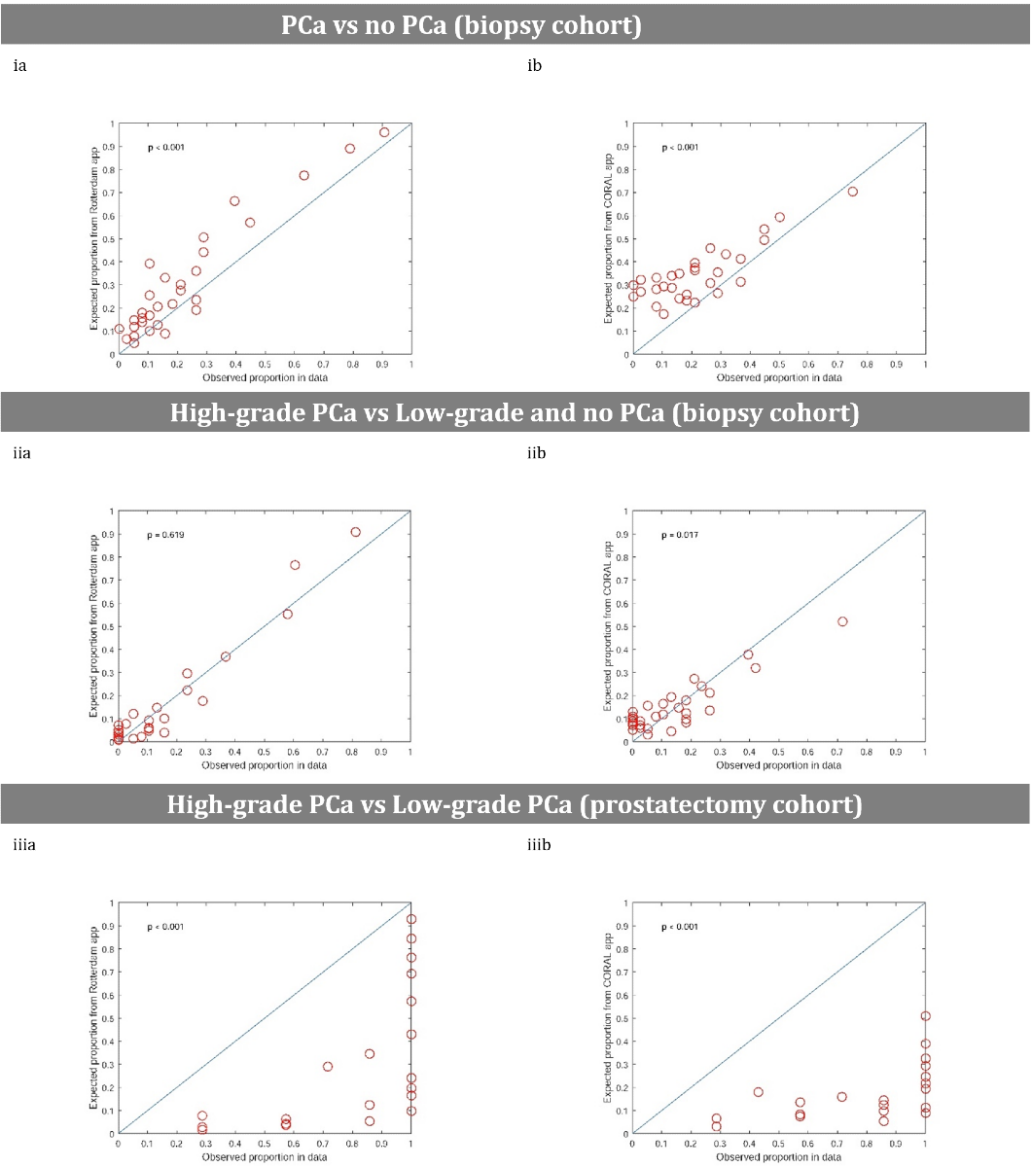
Calibration plots comparing (a) the Rotterdam app and (b) Coral app showing the agreement between (i) predicted and observed probabilities for diagnosing prostate cancer (PCa) and (ii) high-grade PCa in the biopsy cohort, and (iii) high-grade PCa in the prostatectomy cohort. Each circle in the plots represents a group of patients with an observed probability of PCa or high-grade PCa on the x-axis, corresponding to an average calculated risk of PCa or high-grade PCa by the apps on the y-axis. Figures (ia) and (ib) demonstrated overestimation, whereas Figures (iiia) and (iiib) illustrated underestimation. Only Figure (iia) showed a good calibration; in Figure (iib), overestimation was revealed among the lower observed proportions and underestimation among the higher observed proportions.

The calibration of both apps was tested with the Hosmer-Lemeshow goodness-of-fit test (Figure 2). Comparing both apps, only the Rotterdam app demonstrated a good calibration (*P*=.619) for detecting high-grade PCa in the biopsy cohort. Other models were miscalibrated, including all models created from the Coral app.

### Discrimination

**Figure 3:**
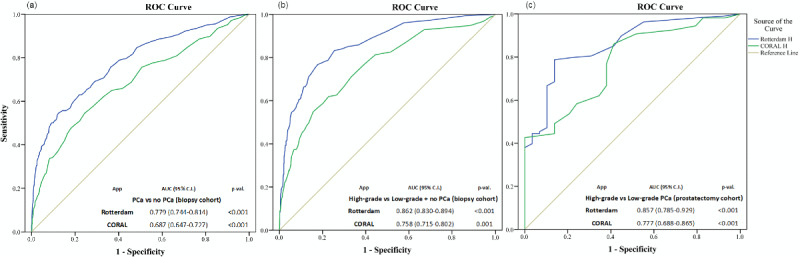
Receiver operating characteristic (ROC) curves and areas under the receiver operating characteristic curves (AUCs) for the discriminative ability of the Rotterdam and Coral apps. (a) Prostate cancer (PCa) vs no PCa in the biopsy cohort; (b) high-grade PCa vs low-grade PCa plus no PCa in the biopsy cohort; (c) high-grade PCa vs low-grade PCa in the prostatectomy cohort.

Both the Rotterdam and Coral apps could significantly predict PCa and high-grade PCa in the biopsy cohort on ROC analysis (Figure 3, a and b). The discriminative capacity for detection of both PCa (AUC: 0.779 vs 0.687; DeLong’s method: *P*<.001) and high-grade PCa (AUC: 0.862 vs 0.758; *P*<.001) was significantly better for the Rotterdam app compared to the Coral app. In the prostatectomy cohort, the Rotterdam and Coral apps were not significantly different for predicting high-grade PCa (AUC: 0.857 vs 0.777; *P*=.128; Figure 3c).

### Clinical Utility

**Figure 4:**
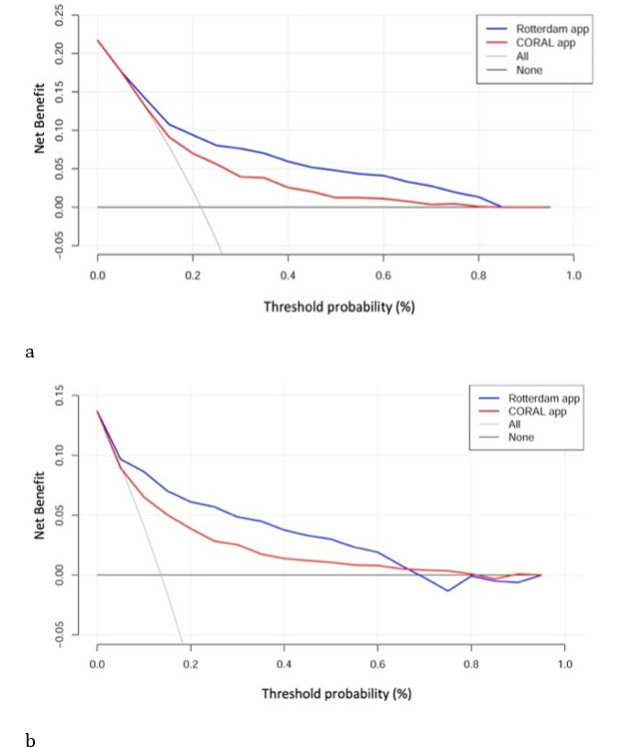
Decision curve analyses quantifying clinical utility by showing the net benefits associated with the use of the Rotterdam app (blue line) and the Coral app (red line) in (a) the detection of prostate cancer (PCa) and (b) high-grade PCa. Decision curves investigate the theoretical net benefit at various threshold probabilities. The oblique gray line assumes that all persons will undergo prostate biopsy, whereas the horizontal black line along the x-axis assumes that no one will receive biopsy. The threshold probability may correspond to the calculated prostate cancer risk. The area under the curve between these 2 lines illustrates net benefits. In the same range of threshold probability, higher net benefits represent better clinical utility.

**Figure 5:**
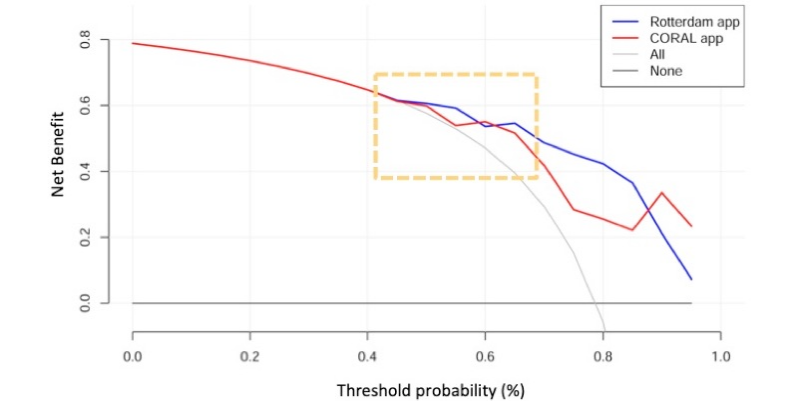
Decision curve analysis showing the net benefits of utilizing the Rotterdam app (blue line) and the Coral app (red line) to detect high-grade prostate cancer (PCa) in the prostatectomy cohort. The curves are skewed because the incidence of high-grade PCa in the prostatectomy cohort is relatively high and the sample size is small. No net benefit could be demonstrated below the risk threshold of 50%.

In the decision curve analysis for the biopsy cohort of patients, both apps demonstrated clinical net benefits in the threshold probability range of 10% to 85% for the detection of any PCa. In the detection of high-grade PCa, the Rotterdam and Coral apps provided net benefits in the threshold probability range of 5%-70% and 10%-80%, respectively. In comparing both apps, the net benefit was greater for the Rotterdam app in the prediction of both PCa and high-grade PCa across the range of threshold probabilities from 5%-70% (Figure 4). It seemed that both apps provided net benefits for the detection of high-grade PCa in the prostatectomy cohort (Figure 5). Nevertheless, the prevalence of high-grade PCa in the prostatectomy cohort was very high (79%). With baseline risk being very high, it would be difficult for both apps to push the risk to a level low enough for advice against biopsy. Both apps had higher net benefits when the curves diverged at the threshold probability of about 50%, and therefore, both apps lacked value for the prediction of high-grade PCa in the prostatectomy cohort.

## Discussion

### Principal Findings

In this study, we found that the Rotterdam and Coral apps were both applicable to the Taiwanese cohort of patients who had undergone TRUS prostate biopsy, even though these apps were built based on Western populations. In order to externally validate these 2 apps, 3 key statistical measures were used in the assessment of predictive performance. Firstly, most models revealed miscalibration, but the Rotterdam app demonstrated good calibration for the prediction of high-grade PCa in the biopsy cohort. Secondly, the Rotterdam app outperformed the Coral app in its discriminative ability for predicting PCa and high-grade PCa in the biopsy cohort. Thirdly, the Rotterdam app provided greater net benefits than the Coral app to assist in biopsy decision-making. In brief, the Rotterdam app delivered better predictive performance than the Coral app for PCa and high-grade PCa in our Taiwanese population cohort.

To the best of our knowledge, at the stage before PCa is diagnosed, no risk prediction model has ever been validated by the whole prostate specimen. Data from the 137 patients who had undergone radical prostatectomy for any-grade PCa were utilized to evaluate the Rotterdam and Coral apps' predictive capacity for high-grade PCa. Both apps demonstrated fairly good discrimination for predicting high-grade PCa in the prostatectomy cohort and the biopsy cohort. It was implied that after confirmation with postprostatectomy pathology outcomes, both apps still delivered a comparable discriminative ability for predicting high-grade PCa in the present Taiwanese cohort. However, during calibration and decision curve analysis in the prostatectomy cohort, both apps were miscalibrated and revealed few net benefits. This might be explained by the small sample size and different pathology distribution, as there was a higher prevalence of high-grade PCa (79%). Moreover, these apps were built on biopsy cohorts, which are different from the prostatectomy cohort. More patients with radical prostatectomy might need to be enrolled to validate app predictability of high-grade PCa, which would be validated by the whole prostate specimen.

In current clinical practice, most patients with an abnormal PSA level of >4 ng/mL or an abnormal DRE are put forward for biopsy. However, such indications lead to a myriad of unnecessary biopsies and associated complications, such as hematuria, hematospermia, rectal bleeding, acute urinary retention, urinary tract infection, or even sepsis. To increase the accuracy of cancer detection and reduce unnecessary postbiopsy morbidities, several biomarker tests have been developed, including the Prostate Health Index (PHI), percent-free PSA, PCA3, 4K-score, etc [14]. The European Association of Urology (EAU) guidelines suggest an individualized evaluation of PCa risk. Age, family history of PCa, DRE, serum or urine markers, and mpMRI are validated parameters in combination with PSA levels to help predict the risk of PCa [15]. Recently, professionals have formulated a number of PCa risk calculators using some of these useful predictors to improve predictive accuracy, and such multivariable risk approaches have performed better than PSA or DRE alone [10]. Nonetheless, most of them have only been validated in independent cohorts; neither superiority nor global applicability has been shown [16]. De Nunzio et al [4] had validated the Rotterdam and Coral apps' discriminative abilities utilizing a southern European cohort as providing better predictive performance than PSA or DRE; however, the predictability of PCa or high-grade PCa in the Taiwanese population stills needs to be addressed.

It is well known that Gleason upgrading occurs in 32%-49% of patients with initial biopsy of low-grade (Gleason 3+3) PCa at the time of pathological assessment of the whole prostate specimen [14]. Verep et al [17] reviewed 137 patients who were eligible for active surveillance but underwent radical prostatectomy at their institution. The criteria of active surveillance included Gleason 3+3 adenocarcinoma, maximum 2 positive biopsy cores, PSA <10ng/mL, and clinical T-stage equal or less than 2a. Following pathological confirmation, Gleason upgrading was noted in almost half of the patients (49.3%), and upstaging to pT3a occurred in 17 patients (12.5%) [17]. Due to the risks of over-diagnosis and over-treatment for clinically insignificant PCa, active surveillance has become increasingly adopted as a preferred treatment option for patients with low-grade PCa. However, without precise risk stratification, active surveillance might delay the timing of curative treatment for localized PCa, or even increase the risks of lymph node involvement and distant metastasis. Consequently, the accuracy of risk prediction tools has become of paramount importance.

Mobile health (mHealth) is regarded as a valuable tool to implement patient-centered care, which is in accordance with the individualized risk assessment of PCa recommended by the EAU guidelines. mHealth can provide access to health information, skills, and services, and can also promote positive health behavioral changes to prevent acute and chronic diseases. Real-time monitoring can obtain live data from patients and transmit inputs to a network or a medical app on a smartphone to assist clinical decision-making. Regardless of the environmental circumstances, geographical barriers, and conventional infrastructures, it can share timely information between patients and health personnel, replacing the traditional face-to-face platform of medical care. Nevertheless, to not harm patients, it is pivotal that scientific accuracy, patient safety, and user privacy of mHealth apps be assured [18].

One systematic review that critically appraised PCa risk calculator apps maintained that the Rotterdam Prostate Cancer Risk Calculator and Coral–Prostate Cancer Nomogram Calculator outperformed other apps [3]. The authors utilized the validated user version of the Mobile Application Rating Scale to individually assess and rate 7 apps, including 3 categories of app quality ratings, subjective quality, and perceived impact [19]. Objective characteristics were thoroughly documented and assessed. None of these apps allowed confidentiality, data storage developing trends, or customization. Both the Rotterdam and Coral apps were found to help differentiate low-grade from high-grade PCa, a noteworthy characteristic of patient counseling in active surveillance compared to other curative alternatives.

Mobile technology enables clinicians and patients to download the Rotterdam and Coral apps readily, and the owners can utilize these apps without an internet connection. They both have the advantages of being less time-consuming and more cost-effective, delivering better immediacy, upgradability, and shareability than the original risk calculators. In addition, they are globally available and recommended by the American Urological Association and EAU guidelines to improve prediction and help determine the risk of PCa stratification. Compared with the Coral app, the conspicuous disadvantage of the Rotterdam app is its cost ($1.99 USD). However, it has been proven to reach a wider audience, with availability on both Apple and Android platforms. While the Coral app has merely 1 language choice, the Rotterdam app has 7 different language options, including Chinese, Dutch, English, Estonian, German, Portuguese, and Spanish.

### Limitations

This study was a single-institution retrospective study, and more cohorts from other Taiwanese hospitals are required to confirm the results. Also, the sample size in the prostatectomy cohort was rather small (n= 137). Notwithstanding that, this is the first study validating PCa risk calculators with postprostatectomy pathological outcomes, although the predictive accuracy of both apps for true high-grade PCa could not be completely determined. Further, no PHI data are available in our institution. The PHI is a combination of 3 blood tests measuring different forms of the PSA protein (total PSA, free PSA, and p2PSA) and calculated as (p2PSA/fPSA) × √tPSA. It is one of the predictors listed in the Rotterdam app, although the risks of PCa can still be calculated without PHI data. Few patients underwent mpMRI before biopsy in our cohort; however, mpMRI has emerged as an important prediction tool to identify clinically significant PCa, especially before a repeated biopsy, which has been recommended by the guidelines [14,15]. The Rotterdam app and web-based ERSPC-RC is one of the PCa risk prediction models incorporating mpMRI (PI-RADS 1-5) [20]; nevertheless, the predictor of the PI-RADS used in the Rotterdam app was the first version, and most of our MRI images were graded according to the PI-RADS v2 guidelines. Moreover, the issues of interobserver variability and heterogeneous definitions of *abnormality* in mpMRI interpretation remain to be explored, and these MRI risk prediction models need to be validated further.

### Conclusions

In our external validation study, the Rotterdam and Coral mHealth apps could be applied to the Taiwanese cohort of patients. Following an assessment of calibration, discrimination, and clinical utility, the Rotterdam app outperformed the Coral app for predicting both any-grade PCa and high-grade PCa. The size of the prostatectomy cohort was small; however, both mobile phone apps, to some extent, demonstrated a predictive capacity for true high-grade PCa, confirmed by the whole prostate specimen. As of yet, there is no PCa risk calculator app developed specifically for the Taiwanese population; however, the Rotterdam app might be a good alternative to enhance the predictive accuracy of current methods for detecting PCa and high-grade PCa.

## Abbreviations

AUC: area under the receiver operating characteristic curve
EAU: European Association of Urology
ERSPC-RC: European Randomized Study of Screening for Prostate Cancer risk calculator
DRE: digital rectal examination
mHealth: mobile health
mpMRI: multiparametric magnetic resonance imaging
MRI: magnetic resonance imaging
PCa: prostate cancer
PCPT: North American Prostate Cancer Prevention Trial
PHI: Prostate Health Index
PI-RADS: Prostate Imaging Reporting and Data System
PSA: prostate-specific antigen
PV: prostate volume
ROC: receiver operating characteristic
TRUS: transrectal ultrasound
